# Integrative analysis of YTHDC1 phosphoproteome unveils its phosphomodulatory network linked to splicing and tumorigenesis

**DOI:** 10.3389/fbinf.2026.1809047

**Published:** 2026-07-06

**Authors:** Megha Shaji, Levin John, Suhail Subair, Jaytha Thomas, Alimath Sambreena, Susmi Varghese, Athira Perunelly Gopalakrishnan, Vaishnavi Gopalakrishnan, Rajesh Krishna, Rajesh Raju, Sowmya Soman

**Affiliations:** 1 Centre for Integrative Omics Data Science, Yenepoya (Deemed to be University), Mangalore, Karnataka, India; 2 Institute for Regeneration and Repair, University of Edinburgh, Edinburgh, United Kingdom; 3 Zulekha Yenepoya Institute of Oncology, Yenepoya Medical College Hospital, Mangalore, Karnataka, India

**Keywords:** mRNA processing, phosphoproteomics, splicing, tumorigenesis, YTHDC1

## Abstract

**Introduction:**

YTH domain-containing protein 1 (YTHDC1) is a nuclear m6A reader with well-known roles in mRNA splicing, nuclear mRNA export, and DNA damage response. Although over 44 phosphosites, including those within the YTH domain, are detected in phosphoproteomic datasets, the phosphoregulatory mechanism that governs YTHDC1 function remains unknown.

**Methods:**

To delineate the phosphoregulatory landscape of YTHDC1, we conducted an integrative analysis of large-scale human phosphoproteomic datasets. The phosphosites were ranked according to their frequency of occurrence, and most recurrent sites were considered predominant. To explore their biological significance, we combined co-phosphorylation analysis with upstream kinase prediction and protein-interaction network mapping.

**Result:**

Three phosphosites, S308 and S146 located in the intrinsically disordered regions and S424 in the YTH domain, were identified as predominantly perturbed across datasets. Functional enrichment analysis of phosphosites in other proteins (PsOPs) co-regulated with these YTHDC1 phosphosites revealed their potential association with mRNA processing and splicing. Considering that no kinases are validated for these sites, phosphomotif-based analysis identified upstream kinases such as MAPK14, CDK7, AKT1, and PAK1. Annotation of phosphosites in these kinases co-regulated with the predominant YTHDC1 phosphosites demonstrated their association with kinase activity, reiterating their potential role as upstream kinases. The PsOPs, including these kinases, as well as many validated binary interactors associated with splicing-related functions, were enriched in the YTHDC1 phosphoregulatory network. Notably, 30 of the co-regulated PsOPs were enriched in pathways that are linked to carcinogenesis, and 4 were in DNA repair inhibition, thereby corroborating their possible role in phosphorylation-dependent signaling associated with cancer.

**Conclusion:**

Considering that targeted molecular biology experiments to explore the role of multiple phosphosites are challenging, our approach provides a suitable framework to infer phospho-site centric regulatory networks. Current findings suggest a putative role of YTHDC1 and its predominant phosphosites in RNA splicing and highlight its regulatory potential in tumor-associated signaling networks.

## 1 Introduction

YTH domain-containing protein 1 (YTHDC1) is a 727-amino acid nuclear m6A reader, encoded at the 4q13.2 locus (Zuo et al., 2024; UniProt, 2025; Hornbeck et al., 2012). N6-methyladenosine (m6A) modification is one of the most prevalent and evolutionarily conserved mRNA modifications observed in eukaryotes and some RNA viruses (Li, 2022a). The methylation pattern of m6A RNA is dynamically regulated by the coordinated interplay of a set of RNA-modifying proteins (RMPs). The RMPs linked to m6A modification consist of writers, erasers, and readers, which correspond to the deposition, removal, and binding of m6A, respectively (Lin et al., 2024).

The human YTH family represents the most well-known m6A readers, which comprises five members: YTH N6-methyladenosine RNA-binding protein 1 (YTHDF1), YTH N6-methyladenosine RNA-binding protein 2 (YTHDF2), YTH N6-methyladenosine RNA-binding protein 3 (YTHDF3), YTH domain-containing 1 (YTHDC1), and YTH domain-containing 2 (YTHDC2) (Liao et al., 2022). YTHDC1 is involved in RNA splicing by interacting with splicing factors (Zuo et al., 2024). It is a nuclear localized protein, making it distinct from YTHDF1-3 and YTHDC2, which are mainly cytoplasmic (Widagdo et al., 2022). In the nucleus, it forms speckled structures known as YT bodies, a subnuclear region linked to active transcription and RNA-processing speckles (Zuo et al., 2024). Previous reports have demonstrated that phosphorylation of YTHDC1 by Src family kinases such as c-src and p59Fyn influenced the solubility and distribution of YTHDC1 in YT bodies, which resulted in nucleoplasmic diffusion and altered interaction of pre-mRNA protein complexes (Li et al., 2024).

YTHDC1 has an internal YTH domain flanked by two intrinsically disordered regions (N-terminal and C-terminal) rich in arginine, proline, aspartate, and glutamate. Conversely, the YTH domains of YTHDF1-3 and DC2 are located within the C-terminal region (Woodcock et al., 2020). YTHDC1 also exhibits a unique sequence-specific binding preference among YTH family proteins, which specifically binds to the GA* motif (guanosine at the −1 position before the methylated adenosine) instead of the AA* motif (Widagdo et al., 2022) and the recognition of m6A is mediated by an aromatic pocket composed of Trp411, Trp465, and Trp470 that specifically accommodates the m^6^A site (Liao et al., 2022).

Through its m6A-dependent interactions, YTHDC1 is associated with multiple RNA metabolic processes, including mRNA splicing, nuclear mRNA export, DNA damage response, transcription and maintaining the stability of m6A-modified RNAs (Ge et al., 2023). In the nucleus, YTHDC1 recognizes methylated mRNAs and regulates their processing and export via nuclear RNA export factor 1 (NXF1) by promoting recruitment of serine and arginine-rich splicing factor 3 (SRSF3) to pre-mRNAs and inhibiting the binding of serine and arginine-rich splicing factor 10 (SRSF10), thereby maintaining efficient mRNA splicing (Xiao et al., 2016; Roundtree et al., 2017).

In addition to RNA metabolic processes, YTHDC1 is also involved in the regulation of some signaling pathways. In inflammatory bowel disease (IBD), YTHDC1 suppresses autophagy-dependent NF-κB signaling by altering the stability of Beclin1 mRNA (Zhou et al., 2024). YTHDC1 inhibits clear cell renal cell carcinoma (CCRCC) by downregulating the ANXA1/MAPK signaling pathways (Li, 2022c). In triple-negative breast cancer, YTHDC1 activates the TGF-β signaling pathway by facilitating nuclear export and the upregulation of SMAD family member 3 (SMAD3) (Tan et al., 2022). The dysregulation of YTHDC1 is associated with the progression of diseases, particularly across various cancers such as triple-negative breast cancer (TNBC), acute myeloid leukemia (AML), ovarian cancer, colon cancer, hepatocellular carcinoma, lung cancer, and glioblastoma (Yan et al., 2022). YTHDC1 overexpression has been reported in TNBC (Tan et al., 2022), colon cancer (Gao et al., 2025), hepatocellular carcinoma (Du et al., 2024), and AML (Sheng et al., 2021), while reduced expression has been reported in ovarian cancer (Wang et al., 2023) and lung cancer (Yuan et al., 2023). YTHDC1 functions as a tumorigenic factor in colon cancer. Its absence results in an elevation of the ubiquitin protein ligase E3A (UBE3A), which activates the ubiquitination of RAD51 recombinase (RAD51) and subsequently triggers apoptosis in cancer cells (Gao et al., 2025). Conversely, YTHDC1 functions as a tumor suppressor in ovarian cancer by enhancing the stability of phosphoinositide-3-kinase regulatory subunit 1 (PIK3R1) mRNA through a m6A-dependent mechanism. This action inhibits neutral alpha-glucosidase AB (GANAB)-mediated N-glycan biosynthesis via signal transducer and activator of transcription 3 (STAT3) signaling (Wang et al., 2023).

Even though it is well known that YTHDC1 plays a role in post-transcriptional regulation and cancer-related signaling pathways, the phosphoregulatory mechanisms that control its activity have not been studied. In particular, the current knowledge on the upstream kinases, downstream effectors, and the interacting partners of YTHDC1 is limited.To address this gap, we employed an integrative phosphoproteomic approach to identify YTHDC1 phosphosites, kinase-mediated pathways and biological processes associated with YTHDC1, thereby integrating YTHDC1 into cellular phosphoregulatory networks. Unlike conventional phosphoproteomic studies that focus on protein-level changes, our approach adopts a phosphosite-centric framework to identify recurrent and co-regulated phosphosrylation events, enabling site-specific functional insights. Overall, this study could provide a foundational framework for the mechanistic and functional role of YTHDC1, which can be utilized for its evaluation using targeted experimental approaches.

## 2 Materials and methods

### 2.1 Systematic phosphoproteomic dataset screening for YTHDC1 phosphosites

To systematically delineate the phosphorylation landscape of YTHDC1, an extensive literature search was carried out in PubMed using the keywords “phosphoproteomics” OR “phosphoproteome”, while excluding “Plant” and “Review”. Our analysis focused only on high-confidence phosphoproteomics datasets obtained by mass spectrometry from human cell lines. The datasets were systematically screened and curated from the previously published studies. These datasets were categorized according to the phosphosite enrichment strategies used in each study (ST/STY/Y phosphosites).

In order to maintain high confidence in the detected phosphosites, only phosphosites with a localization probability of ≥75% and an A-score **≥**13 were considered and defined as Class-1 phosphosites. For further analysis, datasets were classified into quantitative differential datasets, in which phosphosite abundances were compared between experimental/test conditions and their corresponding controls, and qualitative profile datasets, where test and control phosphosite profiles were treated as independent entities.

To identify significant regulatory changes based on the fold change and statistical significance (p < 0.05), differential datasets containing Class-1 phosphosites were further analyzed. Phosphosites were considered as upregulated if their fold changes were ≥1.3, and as downregulated if they were ≤0.76. These thresholds enabled robust and reliable identification of biologically significant phosphorylation events. Individual proteins from each dataset were mapped to their respective gene symbols in accordance with the latest HUGO Gene Nomenclature Committee (HGNC) guidelines (Seal et al., 2023) to make sure consistency across datasets, and phosphosites were systematically mapped to Uniprot accessions (UniProt, 2025) using our in-built mapping tool (Sheela et al., 2025).

### 2.2 Identification of predominant YTHDC1 phosphosites in cellular phosphoproteomes

The Class-1 phosphosites of YTHDC1 were extracted from the human cellular phosphoproteome profile datasets. For each phosphosite, we calculated and ranked the number of qualitative profile datasets in which it was observed or reported, and the number of quantitative differential datasets where it was found to be differentially regulated. Phosphosites identified in more than 50% of qualitative and quantitative profile datasets were considered as predominant phosphosites. Importantly, phosphosites detected using phospho-specific antibodies or mutation-driven methodologies, which were not commonly reported as Class-1 phosphosites in these datasets, were excluded from this analysis.

### 2.3 Identification of protein phosphosites co-differentially regulated with predominant YTHDC1 phosphosites

To identify the phosphosites in other proteins (PsOPs) that exhibit positive and negative co-differential regulation with the predominant YTHDC1 phosphosites (S146, S308, and S424), the differential regulation datasets for each phosphosite were independently classified. Considering the extensive number of datasets covering diverse experimental conditions, biological systems, and distinct analysis platforms, reanalysis of the data was not feasible. Thus, for further analysis, phosphosites with a localization probability **≥**75% and an ambiguity score **≥**13 were included. The following analysis was carried out separately for each predominant YTHDC1 phosphosite. PsOPs (denoted as o) that showed either downregulation or upregulation in association with YTHDC1 (denoted as y) upregulation were denoted as DoUy and UoUy, respectively. Conversely, those exhibiting similar regulation with YTHDC1 downregulation were denoted as DoDy and UoDy. The PsOPs in UoUy and DoDy were regarded as a positive co-regulation, whereas those in DoUy and UoDy were regarded as a negative co-regulation with YTHDC1 expression.

To investigate the co-regulation between the YTHDC1 predominant phosphosites and their associated PsOPs across quantitative differential datasets, a one-sided Fisher’s exact test was carried out using a contingency table.

Fisher’s exact test (FET):

$$\sum P=\frac{\left(a+b\right)!\left(c+d\right)!\left(a+c\right)!\left(b+d\right)!}{n!}\sum_{i}\frac{1}{a_{i}!b_{i}!c_{i}!d_{i}!}$$

where a (n_0y0o) represents the number of experimental conditions in which neither the YTHDC1 phosphosites nor the PsOPs were detected; b (n_Uy0+n_Dy0+n_0Uo + n_0Do) denotes the number of experimental conditions in which only one of the two phosphosites was detected (either up- or downregulated), while the other was absent; c (n_UyDo + n_DyUo) corresponds to the number of experimental conditions exhibiting negative regulation between the two phosphosites; d (n_UyUo + n_DyDo) represents the number of experimental conditions showing positive regulation. Using these values, a contingency table was generated, and a one-sided Fisher’s exact test was performed to determine the statistical significance of co-regulation (Kammarambath et al., 2025).

Potential biases in cellular phosphoproteomic datasets, such as overrepresentation of multi-temporal datasets and datasets generated under similar experimental stimuli, were taken into account. To mitigate these biases, datasets were filtered based on four criteria including a FET p-valu <0.05; a minimum threshold of 10% for the ratio of n (UyUoDyDo)/(UyDoDyUo) and n (UyDoDyUo)/(UyUoDyDo) to distinguish positively and negatively co-differentially regulated phosphosite pairs respectively, thereby ensuring consistent regulation pattern across datasets; a minimum of threeseparate experimental conditions that should indicate positive or negative regulation; and a minimum of three independent publications (PubMed IDs) that support positive or negative regulation (Lalu et al., 2025). Only the PsOPs satisfying these criteria were classified as high-confidence and used for further analysis.

### 2.4 Identification of upstream kinases and binary interactors of YTHDC1

Various computational methodologies were employed to identify the upstream kinases of YTHDC1 predominant phosphosites. The kinases regulating YTHDC1 phosphorylation were catalogued using the protocol of Johnson et al. (2023), which involves synthetic peptide screening to determine kinome-wide substrate specificity, with a 90th percentile cutoff. Experimentally validated kinases and their corresponding phosphosites of YTHDC1 predominant phosphosites were obtained from curated databases, including PhosphoSitePlus (Hornbeck et al., 2015), RegPhos 2.0 (Huang et al., 2014), and Phospho. ELM (Dinkel et al., 2011). In addition, kinases predicted using NetworKIN (Linding et al., 2008), and AKID (Parca et al., 2019) were also included for the analysis. Experimentally identified protein-protein interactors of YTHDC1 were gathered by integrating data from multiple sources, including BIND (Bader et al., 2003), HPRD (Keshava Prasad et al., 2009), BioGRID (Oughtred et al., 2021), and ConsensusPathDB (Oughtred et al., 2021; Kamburov and Herwig, 2022; Thomas et al., 2025)**.**

### 2.5 Data visualization

To represent the data, lollipop plots were generated using the R/Bioconductor package track Viewer (10.18129/B9. bioc.track-Viewer). Python packages such as Matplotlib and Pandas were used for the visualization of phosphosite distribution across quantitative differential profile data. Sequence conservation analysis was performed by using Clustal Omega (Sievers and Higgins, 2018). The peptide mapping and alphafold2 predicted structure of YTHDC1 were visualized using sequence coverage visualizer (SCV) (Shao et al., 2023). RAWGraphs 2.0 (https://app.rawgraphs.io/) was used to create a dendrogram. Cytoscape (Shannon et al., 2003) was used for visualizing the interaction network.

## 3 Result and discussion

To investigate the phosphocentric regulatory landscape of YTHDC1, we carried out an integrative analysis of large-scale human phosphoproteomics datasets. This section provides a comprehensive mapping of the YTHDC1 phosphosites and phosphopeptides, their frequency across datasets, and potential functional implications.

### 3.1 Global phosphoproteomic exploration of YTHDC1 and mapping of phosphopeptides and predominant phosphosites

**FIGURE 1:**
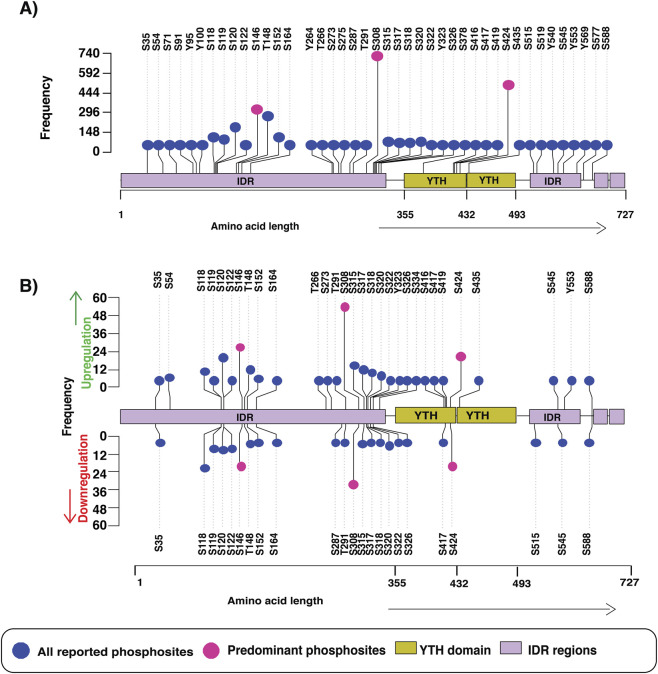
Lollipop plot depicting the phosphosite distribution of YTHDC1. **(A)** Class 1 YTHDC1 phosphosites across 938 profiling datasets. **(B)** Class 1 YTHDC1 phosphosites identified in 198 differential datasets.

To identify phosphorylation events associated with YTHDC1 phosphorylation, we screened over 3,825 publicly available human global phosphoproteomics articles. This resulted in the retrieval of 938 profiling datasets from 173 PubMed IDs and 198 differential datasets from 64 PubMed IDs, reporting Class 1 YTHDC1 phosphosites (Figure 1). Detailed information on all identified Class 1 YTHDC1 phosphosites from qualitative profiling and quantitative differential datasets are provided in Supplementary Table S1 and a list of profiling and differential datasets and their sources along with respective PubMed IDs, is provided in Supplementary Table S1. Through systematic mapping, 44 YTHDC1 phosphosites were identified from the profiling data, and 32 were identified from the differential data. Among the differentially regulated phosphosites, S308, S146, and S424 were the most frequently regulated, with frequencies of 89, 50, and 44, respectively, and were designated as the predominant phosphosites of YTHDC1.

To explore whether the regulatory significance of these sites is unique to YTHDC1 or common to the YTH-domain containing family, we assessed the evolutionary conservation of these predominant phosphosites using Clustal Omega. None of the predominant phosphosites were evolutionarily conserved, suggesting that the regulatory modifications were unique to YTHDC1 rather than a common feature of the YTH-domain containing protein family. Mapping these phosphosites onto the domain architecture of YTHDC1 revealed that S424 resides within the YTH domain, whereas S308 and S146 are positioned within the intrinsically disordered region (IDR). Prior reports on IDRs suggest that these regions are highly flexible within the proteins that are prone to conformational dynamics and can serve as crucial regulatory hubs (Singleton and Eisen, 2024). The phosphorylation within the IDR is often associated with the dynamic regulation of protein interaction and signaling responses (Modic et al., 2024). These findings suggest that phosphorylation at sites such as S308 and S146 may modulate conformation, interaction, and functional activity of YTHDC1. In contrast, phosphorylation within the YTH domain may influence RNA binding properties. Given that the YTH domain mediates m6A recognition, modification within this region could influence binding affinity and specificity. The identification of predominant phosphosite S424 within this domain suggests that its phosphorylation might affect m6A-dependent RNA recognition and subsequent RNA processing events. However, these interpretations remain hypothesis-generating and require experimental validation.

#### 3.1.1 Peptide coverage and phosphosite mapping of YTHDC1

**FIGURE 2:**
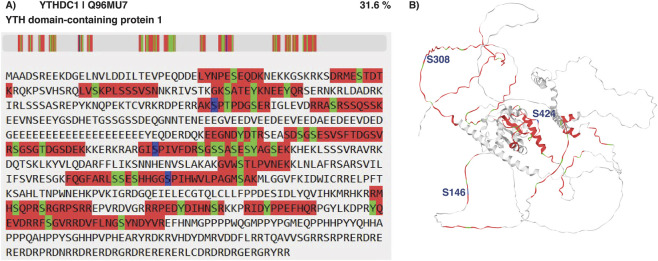
Sequence coverage and phosphosite mapping of YTHDC1 using SCV. **(A)** Linear sequence of YTHDC1 showing 31.6% peptide coverage from profile datasets. **(B)** Alphafold predicted 3D structure of YTHDC1, highlighting peptides identified across datasets. Sequence coverage is shown in red, Ser/Thr/Tyr phosphosites are indicated in green, and the predominant phosphosites are highlighted in blue.

Across the 938 profile datasets with YTHDC1 phosphosites, 42 unique phosphorylated peptides covering 31.6% of its total 727 amino acid residues were reported. All these phosphopeptides were detected between residues 35 and 588. This hyperphosphorylated region indicates the potential for dynamic structural conformation that may enable interaction and further regulatory involvement. Phosphopeptides corresponding to S308, S146, and S424 predominant phosphosites were observed in 735, 332, and 489 datasets, respectively. The total peptide coverage of the YTHDC1 and its alphafold predicted structure is illustrated in Figure 2. To further explore the regulatory patterns associated with YTHDC1, we explored the positively and negatively co-regulated PsOPs of predominant phosphosites.

### 3.2 Co-regulated phosphosites associated with YTHDC1 predominant phosphosites

**FIGURE 3:**
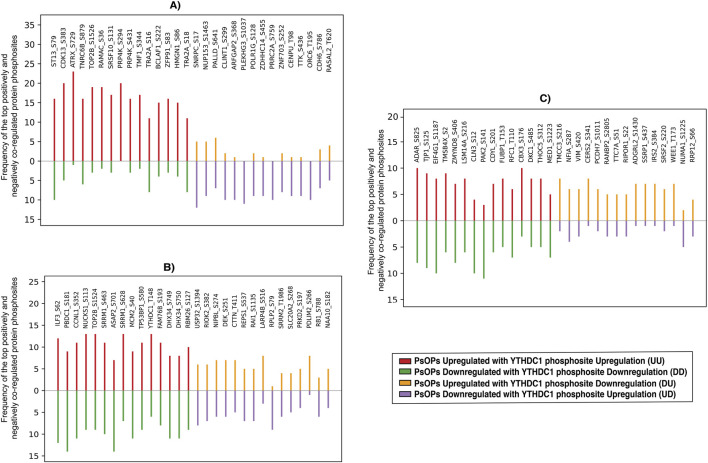
Bar plot representing the top 15 phosphosites in other proteins that showed the highest frequency of positive and negative co-regulation with respect to YTHDC1 predominant phosphosites **(A)** S308, **(B)** S146 and **(C)** S424.

In order to gain deeper insights into the phosphoregulatory networks associated with the predominant phosphosites of YTHDC1, a co-regulation analysis was carried out based on the pairwise expression of the predominant phosphosite and PsOPs that are present in the datasets. The PsOPs, which show co-regulation with the predominant YTHDC1 sites, were fetched using a set of stringent criteria described in the methodology section. This resulted in the identification of high-confidence co-regulated proteins. The high-confidence PsOPs that are co-regulated with predominant phosphosites of YTHDC1 are provided in Supplementary Table S2. Based on the selection criteria, we identified 823 positively and 27 negatively co-regulated PsOPs associated with YTHDC1 (S308). Similarly, 1682 positively and 139 negatively co-regulated PsOPs were detected for YTHDC1 (S146), and 595 positively and 101 negatively co-regulated PsOPs for YTHDC1 (S424). The top 15 high-confidence PsOPs that are positively or negatively co-regulated with YTHDC1 predominant phosphosites are represented in Figure 3.

Within the high-confidence set of PsOPs associated with YTHDC1 (S308), ST13 Hsp70 interacting protein (ST13) (S79) exhibited the highest positive co-regulation, whereas Small nuclear ribonucleoprotein polypeptide C (SNRPC) (S17) showed the highest negative co-regulation. Likewise, among the PsOPs co-regulated with YTHDC1 (S146), interleukin enhancer-binding factor 3 (ILF3) (S62) showed the highest positive co-regulation, whereas ubiquitin-specific protease 32 (USP32) (S1394) showed the highest negative co-regulation. For YTHDC1 (S424), adenosine deaminase acting on RNA (ADAR) (S825) and transmembrane and coiled-coil domain family 3 (TMMC3) (S216) exhibited the highest positive and negative co-regulation, respectively. Notably, SNRPC, ILF3, and ADAR are functionally involved in RNA splicing and mRNA processing (Cai et al., 2021; Watson et al., 2020; Solomon et al., 2013), thus emphasizing the potential to have a mutual functional correlation with YTHDC1. Specifically, ADAR is associated with splicing through both RNA editing-dependent and RNA editing-independent mechanisms (Weng et al., 2023; Sagredo et al., 2025), whereas ILF3 contributes to various stages of the gene expression pathway, mediating the transcription to translation through RNA splicing, stability, and export (Watson et al., 2020).

### 3.3 Functional characterization of the predominant phosphosites of YTHDC1 and its co-regulated protein network

**FIGURE 4:**
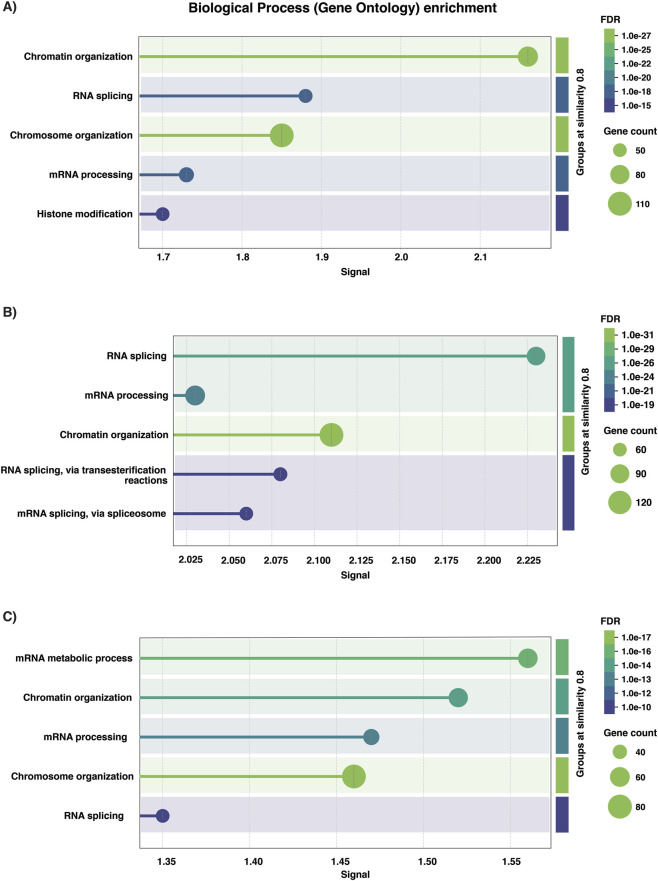
Gene ontology enrichment analysis representing top biological processes associated with PsOPs positively co-regulated with YTHDC1 predominant phosphosites **(A)** S308, **(B)** S146 and **(C)** S424.

To investigate the biological functions associated with predominant phosphosites of YTHDC1 and its co-regulated proteins, we performed a gene enrichment analysis on the positively co-regulated PsOPs corresponding to each predominant phosphosite of YTHDC1 using the STRING database (Szklarczyk et al., 2025). Across all three phosphosites, the most significantly enriched biological process categories were consistently related to RNA splicing, mRNA processing, and chromatin organization (Figure 4).

For the predominant phosphosite S308, chromatin organization emerged as the top-enriched biological process among the five most significant categories. The top positively co-regulated PsOPs associated with chromatin organization include alpha thalassemia intellectual disability syndrome X-linked (ATRX) (S729, S1348), DNA topoisomerase 2-beta (TOP2B) (S1526, S1424), chromobox homolog 8 (CBX8) (S311), bromodomain-containing 4 (BRD4) (S1117), and several spliceosome regulators. These processes are functionally interconnected, as chromatin organization and transcriptional regulation are known to influence co-transcriptional RNA splicing through modulation of spliceosome recruitment and RNA polymerase II dynamics (Yustis et al., 2024).

Similarly, the predominant phosphosite S146 also exhibited positive co-regulation with several splicesomes and splicing regulators, including ILF3 (S62), serine and arginine repetitive matrix 1 (SRRM1) (S463, S628), DEAH-Box helicase 34 (DHX34) (S749, S750). Although similar functions, such as chromatin organization and splicing, were included, the prominent biological process associated with S424 was mRNA processing. Factors such as ADAR (S825), eukaryotic translation initiation factor 4 gamma 1 (EIF4G1) (S1187), LSM14 homolog A (LSM14A) (S216), far upstream element-binding protein 1 (FUBP1) (T153) were consistently represented with positively co-regulated PsOPs. This further highlights the importance of exploring the phosphorylation landscape of YTHDC1 predominant phosphosites.

To further expand these findings, an independent gene enrichment analysis was performed using Enrichr (Kuleshov et al., 2016). This analysis revealed that PsOPs that positively co-regulated with predominant phosphosites of YTHDC1 were mainly associated with mRNA processing and splicing-related biological functions (Supplementary Figure S1). Collectively, these results underscore the functional significance of phosphorylation at the predominant phosphosites of YTHDC1 and highlight its potential role in mRNA-associated biological processes.

### 3.4 Predicted upstream kinases regulating YTHDC1 phosphorylation

Although several phosphosites on YTHDC1 have been identified, the upstream kinases responsible for these phosphorylation events remain unknown due to the lack of direct experimental validation. To address this, we used computational kinase prediction tools such as NetworKIN/AKID and the sequence-based specificity analysis described by Johnson et al. (2023) (Johnson et al., 2023) to infer potential upstream kinases that are responsible for the phosphorylation of YTHDC1.

**FIGURE 5:**
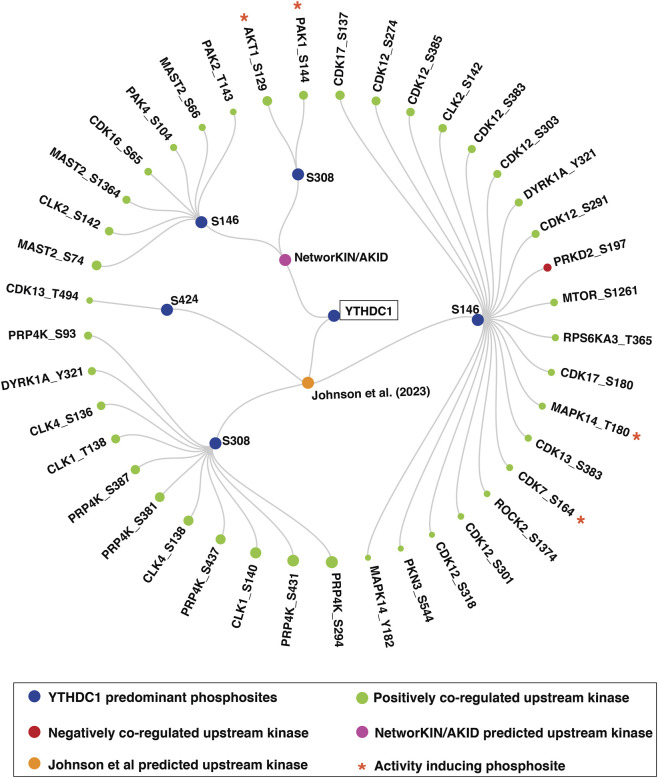
Network representation of predicted upstream kinases of YTHDC1 predominant phosphosites (S308, S146, and S424). Blue colour nodes represent predominant phosphosite of YTHDC1, orange colour node represents the upstream kinase predicted by Johnson et al. (2023), Pink colour node represents upstream kinases predicted by NetworKIN/AKID, green node represents positively co-regulated upstream kinases and red colour node represents negatively co-regulated upstream kinases. Edges represents co-regulation between predominant phosphosites of YTHDC1 and PsOPs. The variation in node size indicates the frequency of co-regulated upstream kinases, where larger nodes corresponds to phosphosite observed more frequently across datasets.

In total, 15 kinases were predicted as unique among the PsOPs co-regulated with predominant phosphosites of YTHDC1 based on Johnson et al. (2023) and 7 kinases were predicted using NetworKIN/AKID. Upstream kinases co-regulated with predominant phosphosites of YTHDC1 are provided in Supplementary Table S3A. The kinases that are positively or negatively co-regulated with YTHDC1 predominant phosphosites are represented in Figure 5.

Among the Johnson et al. predicted kinases, pre-mRNA processing factor 4 kinase (PRP4K) (S294, S431, S437, S381, S387, S93), CDC-like kinase 1 (CLK1) (S140, T138), CDC-like kinase 4 (CLK4) (S138, S136), and dual-specificity tyrosine phosphorylation-regulated kinase 1A (DYRK1A) (Y321) exhibited positive co-regulation with YTHDC1 (S308). Notably, these kinases are well-established regulators of splicing. Specifically, DYRK and CLK kinases phosphorylate a wide range of substrates involved in RNA splicing, signalling pathways, chromatin-associated transcription, and DNA repair (Lindberg and Meijer, 2021). In particular, PRP4K predominantly phosphorylates splicing factors (Habib et al., 2022), highlighting its possible role in phosphorylation-dependent regulation of alternative splicing.

Consistent with these observations, YTHDC1 (S146) have showed a positive co-regulation with several splicing associated kinases, including cyclin dependent kinase 12 (CDK12) (S274, S385, S383, S303, S291, S301, S318), CDC-like kinase 2 (CLK2) (S142), DYRK1A (Y321), cyclin-dependent kinase 13 (CDK13) (S383) and cyclin dependent kinase 7 (CDK7) (S164). Previous studies have implicated the involvement of these kinases in RNA splicing and transcriptional regulation (Naro et al., 2021; Lindberg and Meijer, 2021). In addition, the kinases such as mitogen-activated protein kinase 14 (MAPK14) (T180, Y182) and CDK7 (S164) were involved in enzymatic activity, thus suggesting a potential functional link between YTHDC1 predominant phosphosites and kinase signalling pathways. Additionally, CDK13 (T494), a known splicing regulator, was also positively co-regulated with the YTHDC1 (S424) phosphosite.

Collectively, this analysis further highlights the phosphoregulatory network of upstream kinases and YTHDC1 phosphosites that are involved with RNA splicing and chromatin organization. Importantly, several predicted kinases such as protein kinase B **(**AKT1) (S129) and P21 activated kinase 1 (PAK1) (S144) were associated with S308 and are known to influence enzymatic activation, whereas microtubule-associated Serine/Threonine kinase 2 (MAST2) (S74, S1364, S66), CLK2 (S142), cyclin-dependent kinase 16 (CDK16) (S65), P21 activated kinase 4 (PAK4) (S104), and P21 activated kinase 2 (PAK2) (T143) were positively co-regulated with S146. Interestingly, among the predicted kinases, DYRK1A was common to the YTHDC1 predominant phosphosites S308 and S146, whereas CDK13 was shared between S146 and S424, both of which are established regulators of RNA splicing. Overall, these findings highlight kinases whose phosphorylation is strongly co-regulated with YTHDC1 phosphosites, suggesting a phospho-signaling network linked to splicing-related signaling pathways.

### 3.5 Phosphosites in binary interactors co-regulate with predominant phosphosites of YTHDC1

**FIGURE 6:**
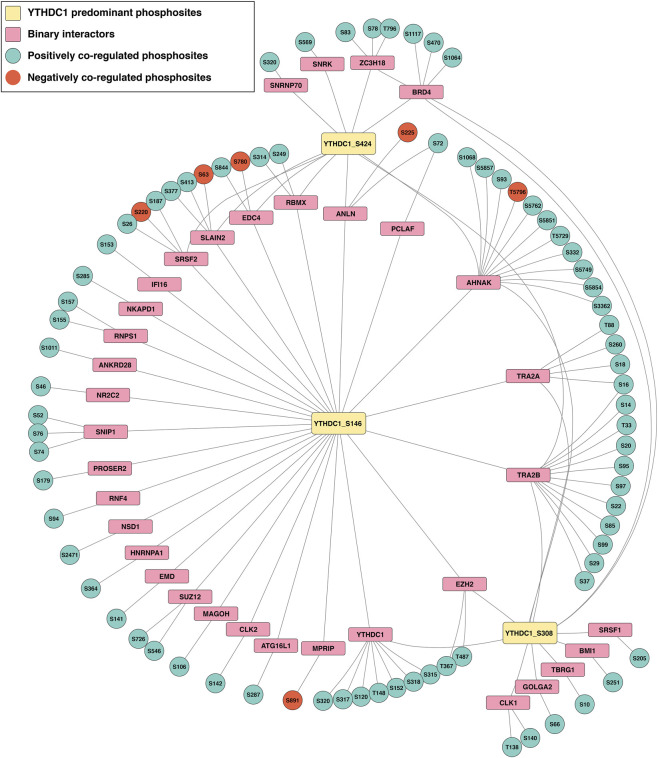
Binary interactors of YTHDC1 that are co-regulated with YTHDC1 predominant phosphosites. Yellow node represents predominant phosphosites of YTHDC1, Pink rectangular nodes represent binary interactors co-regulated with YTHDC1 predominant phosphosites, Teal green circular nodes represent positively co-regulated phosphosites, and orange circular nodes represent negatively co-regulated phosphosites. Edges indicate co-regulation between predominant phosphosites of YTHDC1 and PsOPs.

Protein-protein binary interactions are crucial for understanding the structural and functional organization (Zhang et al., 2015). To investigate the phosphorylation-dependent co-ordination between YTHDC1 and its interactors, we analyzed the PsOPs that are known binary interactors of YTHDC1 using curated databases, including HPRD, BIND, BioGRID, and RegPhos 2.0 (Figure 6). Binary interactors co-regulated with predominant phosphosites of YTHDC1 are provided in Supplementary Table S3B.

For the YTHDC1 (S308), 12 binary interactors were found to be positively co-regulated. Among these, transformer 2 alpha homolog (TRA2A) (S16, S18), transformer 2 beta homolog (TRA2B) (S14, S22), CLK1 (S140, T138), serine and arginine-rich splicing factor 1 (SRSF1) (S205), zinc finger CCCH domain -containing protein 18 (ZC3H18) (S78) are associated with mRNA splicing (Shkreta et al., 2024; Das and Krainer, 2014; Chen et al., 2025). TRA2A and TRA2B, which belong to the serine/arginine-rich (SR) protein family, play a central role in regulating alternative splicing and display frequent dysregulation in many tumor types (Xue et al., 2023). The involvement suggests that YTHDC1 could influence cancer-associated splicing by modulating SR protein phosphorylation and its subsequent activity. This can be supported by previous evidence showing that YTHDC1 regulates oncogenic splicing through the CLK1-SRSF1 axis, where it binds androgen receptor variant 7 (AR-V7) pre-mRNA and facilitates SRSF1 phosphorylation by regulating CLK1 expression, which contributes to the progression of castration-resistant prostate cancer (Gupta et al., 2025). The co-regulation of these proteins suggests a strong co-ordinated phosphorylation dynamics that links YTHDC1 (S308) and splicing-associated complexes.

Similarly, YTHDC1 (S146) also exhibited extensive co-regulation with splicing-related proteins. Among the 26 positively and 2 negatively co-regulated binary interactors, splicing-associated proteins includes TRA2B (T33, S99, S37, S97, S29, S20, S85, S95 and S16), serine and arginine-rich splicing factor 2 (SRSF2) (S187 and S26), CLK2 (S142) and heterogeneous nuclear ribonucleoprotein A1 (HNRNPA1) (S364) (Xue et al., 2023; Li and Wang, 2021; Yoshida et al., 2015; Feng et al., 2022).

For YTHDC1 (S424), 8 positively and 4 negatively co-regulated binary interactors were found. Among these positively co-regulated proteins, ZC3H18 (T796), RNA binding motif protein X-linked (RBMX) (S249), small nuclear ribonucleoprotein U1 subunit 70 (SNRNP70) (S320), TRA2B (S29, T33) are splicing-associated, whereas SRSF2 (S220) represents a negatively co-regulated splicing-related protein (Chen et al., 2025; de Breyne et al., 2025; Nikolaou et al., 2022; Roberts et al., 2014; Li and Wang, 2021). Together, these observations suggest YTHDC1 phosphorylation could influence the regulation of splicing-related factors, thereby underscoring its role in RNA processing.

In conclusion, the above findings indicate a significant link between the co-ordination of the splicing-related protein network and the predominant phosphosites of YTHDC1. The phosphosite-specific differences in the co-regulation of binary interactors further highlight the site-specific regulatory functions in phospho-dependent protein-protein interaction networks within the RNA processing pathways. Dysregulation of RNA splicing can cause several diseases, particularly cancer. Thus, we examined the frequency and regulation of YTHDC1 phosphosites in different datasets across cell lines to assess their potential involvement in tumor-associated signaling pathways.

### 3.6 The role of YTHDC1 in cancer

YTHDC1 exhibits contrasting roles in tumorigenesis. Previous studies have shown that it acts as an oncogene in certain cancers, including TNBC (Tan et al., 2022), colon cancer (Gao et al., 2025), AML (Sheng et al., 2021), while functioning as a tumor-suppressor in lung cancer (Yuan et al., 2023), ovarian cancer (Wang et al., 2023), and CCRCC (Li, 2022c). To delineate its regulation at the post-translational level, we analyzed our curated phosphoproteomic differential datasets across multiple human cell lines, including embryonic stem cells, fibroblasts, cancer cell lines, and transformed cell lines. Among the various cell lines analyzed, cancer cell lines were the most frequent for all three predominant phosphosites. Specifically, S308 was found in 63 of 89 datasets, S146 in 44 of 50 datasets, and S424 in 30 of 44 datasets. The higher occurrence of phosphorylation events in cancer cell lines compared with other cell types suggests that there may be a potential association between YTHDC1 phosphorylation and tumorigenesis/tumor progression. Cancer-specific distribution of YTHDC1 phosphosites across differential datasets were provided in Supplementary Table S4.

**FIGURE 7:**
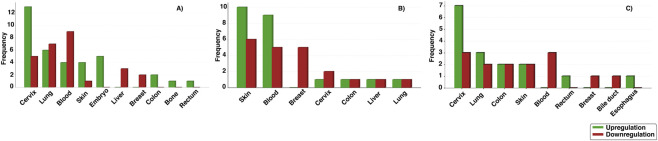
Bar plot depicting the site-specific phosphorylation pattern of YTHDC1 across distinct cancers. The frequency of upregulated and downregulated phosphorylation events is shown for three predominant phosphosites: **(A)** S308, **(B)** S146, and **(C)** S424.

As illustrated in Figure 7, phosphorylation of YTHDC1 shows a distinct pattern across different cancer types in a site-specific manner. The S308 phosphosite emerged as the most frequently identified site among the three predominant phosphosites in cancer cell line data, with the highest representation in cervical cancer cell lines, followed by lung, blood, and skin cancers. In most of these cancer types, S308 is observed mainly as an upregulated phosphorylation event, although cases of downregulation are also evident, reflecting tumor-specific differences. The enrichment in cervical cancer aligns with reports showing reduced YTHDC1 expression in cervical cancer, where YTHDC1 suppresses proliferation, angiogenesis, and metastasis by regulating m6A modification of suppressor of cytokine signaling 4 (SOCS4) mRNA (Chen et al., 2023), suggesting that phosphorylation at S308 may modulate this tumor suppressive pathway. In lung cancer, YTHDC1 has been reported to promote tumorigenesis, where nuclear aurora kinase A regulates YTHDC1 and enhances oncogenic RNA splicing of tumor suppressor RNA binding motif protein 4 (RBM4) (Li, 2022b).

In contrast, phosphorylation at S146 is detected in a smaller subset of cancer cell lines with higher frequencies in skin and blood cancer cell lines and exhibits both up- and downregulated patterns across different malignancies. The S424 site is less broadly distributed but shows a consistent tendency towards upregulation, particularly in cervical and lung cancer cell lines. Given that S424 lies within the YTH domain responsible for m6A recognition, its recurrent upregulation across cancer suggests a potential role in modulating m6A-dependent RNA binding in tumor cells.

The predominant phosphosites of YTHDC1 displayed a distinct pattern of PsOPs across different cell types. Predicted to be involved in functions such as mRNA splicing, mRNA processing, and chromatin organization, YTHDC1 (S308) is positively co-regulated with BRCA1 DNA repair-associated (BRCA1) (S1524) and tumor protein p53 binding protein 1 (TP53BP1) (T543) that are known to promote carcinogenesis and inhibit DNA repair. Similarly, YTHDC1 (S146) positively co-regulated with carcinogenesis-inducing PsOPs such as MAPK14 (T180 and Y182), minichromosome maintenance complex component 2 (MCM2) (S40), and DNA repair-inhibiting PsOPs like mutL homolog 1 (MLH1) (S477), while YTHDC1 (S424) is co-regulated with nibrin (NBN) (S343), tumor protein p53 (TP53) (S315), a key mediator of DNA damage response and cancer progression, highlighting a potential role of YTHDC1 predominant phosphosites in tumor-associated pathways. Consistent with previous reports, YTHDC1 directly regulates TP53 expression by facilitating transcriptional elongation of TP53 and other DNA-damage-associated genes, thereby co-ordinating the DNA damage response and maintaining genomic stability. Disruption of YTHDC1 function leads to defective p53 signaling, genomic instability, and tumor progression (Elvira-Blázquez et al., 2024). Collectively, these findings suggest that the predominant phosphosites of YTHDC1 and the co-regulated PsOPs demonstrated a potential role in tumorigenesis and DNA-repair inhibitory signaling through mRNA processing and splicing. Further experimental studies will be required to determine whether these phosphorylation events have a direct role in tumorigenesis/tumor progression.

## 4 Limitations of the study

This study offers valuable insights into YTHDC1 phosphorylation through the integration of the mass spectrometry-based phosphoproteomic datasets. However, certain limitations should be acknowledged. Integrating large-scale publicly available phosphoproteomics datasets exhibit heterogeneity in data due to differences in various mass spectrometry platforms, experimental conditions, sample types, and data quality. Due to the extensiveness of the curated datasets, limited access to the raw data and a unified reprocessing pipeline the reanalysis of the data for normalisation and batch effect correction was not feasible. To minimize this, we used multiple stringent criteria like FET p-value, experimental code confidence and PMID (PubMed ID) confidence. Though, we used various selection criteria to identify high-confidence co-regulated phosphosites, their functional significance needs to be experimentally validated by site-directed mutagenesis or *in vitro* kinase assays. The findings of our study offer a new venue for future wet-lab studies and provide a framework for phosphosite-specific analysis to advance understanding of signaling pathways and support the development of targeted therapies.

## 5 Conclusion

Our study demonstrated the first comprehensive mapping of YTHDC1 phosphorylation and provided the possible functional associations of the predominant phosphosites S308, S146, and S424. Each predominant phosphosite of YTHDC1 is co-regulated with several PsOPs that are involved in RNA splicing and cancer-associated signalling pathways. This work explores the phosphoregulatory network of YTHDC1 predominant phosphosites, along with the potential upstream kinases and protein-interacting networks.

The enrichment of the YTHDC1 predominant phosphosites in cancer cell lines, and their association with co-regulated proteins that induce carcinogenesis and inhibit DNA repair, underscores their potential role in tumorigenesis. Overall, these findings highlight YTHDC1 phosphorylation as a key regulatory node linking RNA splicing to oncogenic signaling pathways and may serve as a promising phospho-centric biomarker and therapeutic target in cancer. These findings need further experimental validation to establish the functional relevance of YTHDC1 predominant phosphosites.

## Data Availability

The original contributions presented in the study are included in the article/Supplementary Material, further inquiries can be directed to the corresponding author.

## Supplementary material

SUPPLEMENTARY FIGURE S1 The gene enrichment network highlighting the biological processes and associated PsOPs positively co-regulated with YTHDC1 predominant phosphosites: **(A)** S308, **(B)** S146, **(C)** S424. Pink-coloured nodes represent the biological processes associated with the PsOPs. Green colour nodes represent the PsOPs co-regulated with predominant phosphosites of YTHDC1. Edges represent their regulatory relationships.
